# Low levels of taurine introgression in the current Brazilian Nelore and Gir indicine cattle populations

**DOI:** 10.1186/s12711-015-0109-5

**Published:** 2015-04-17

**Authors:** Ana M Perez O’Brien, Daniela Höller, Solomon A Boison, Marco Milanesi, Lorenzo Bomba, Yuri T Utsunomiya, Roberto Carvalheiro, Haroldo HR Neves, Marcos VB da Silva, Curtis P VanTassell, Tad S Sonstegard, Gábor Mészáros, Paolo Ajmone-Marsan, Fernando Garcia, Johann Sölkner

**Affiliations:** Department of Sustainable Agricultural Systems, University of Natural Resources and Life Sciences (BOKU), Vienna, Austria; Università Cattolica del Sacro Cuore di Piacenza, Institute of Zootechnica and Biodiversity and Ancient DNA Research Center - BioDNA, Piacenza, Italy; Universidade Estadual Paulista (UNESP), Faculdade de Ciências Agrária e Veterinárias, Jaboticabal, SP Brazil; Empresa Brasileira de Pesquisa Agropecuária - Embrapa Gado de Leite, Juiz de Fora, MG Brazil; United States Department of Agriculture - Agricultural Research Service, Bovine Functional Genomics Laboratory, Beltsville, MD USA; Universidade Estadual Paulista (UNESP) - Faculdade de Medicina Veterinária de Araçatuba, Araçatuba, SP Brazil

## Abstract

**Background:**

Nelore and Gir are the two most important indicine cattle breeds for production of beef and milk in Brazil. Historical records state that these breeds were introduced in Brazil from the Indian subcontinent, crossed to local taurine cattle in order to quickly increase the population size, and then backcrossed to the original breeds to recover indicine adaptive and productive traits. Previous investigations based on sparse DNA markers detected taurine admixture in these breeds. High-density genome-wide analyses can provide high-resolution information on the genetic composition of current Nelore and Gir populations, estimate more precisely the levels and nature of taurine introgression, and shed light on their history and the strategies that were used to expand these breeds.

**Results:**

We used the high-density Illumina BovineHD BeadChip with more than 777 K single nucleotide polymorphisms (SNPs) that were reduced to 697 115 after quality control filtering to investigate the structure of Nelore and Gir populations and seven other worldwide populations for comparison. Multidimensional scaling and model-based ancestry estimation clearly separated the indicine, European taurine and African taurine ancestries. The average level of taurine introgression in the autosomal genome of Nelore and Gir breeds was less than 1% but was 9% for the Brahman breed. Analyses based on the mitochondrial SNPs present in the Illumina BovineHD BeadChip did not clearly differentiate taurine and indicine haplotype groupings.

**Conclusions:**

The low level of taurine ancestry observed for both Nelore and Gir breeds confirms the historical records of crossbreeding and supports a strong directional selection against taurine haplotypes via backcrossing. Random sampling in production herds across the country and subsequent genotyping would be useful for a more complete view of the admixture levels in the commercial Nelore and Gir populations.

**Electronic supplementary material:**

The online version of this article (doi:10.1186/s12711-015-0109-5) contains supplementary material, which is available to authorized users.

## Background

Brazil has the second largest bovine population in the world [1] with more than 211 million heads of cattle as of 2012 [2], from which about 80% are estimated to be indicine cattle (*Bos primigenius indicus*) [3]. In the last decade, Brazil has emerged as one of the top beef exporters in the world and has a pivotal role in contributing towards ensuring protein availability for the growing global population, especially in emerging countries, which have increasing demands for animal products [4]. The Nelore breed has the largest population and is the main breed used for beef production in Brazil [5]. The Gir breed represents about 10% of indicine cattle and is recognized as the indicine breed with the highest dairy capacity, which has favored its use in recent years [6]. A better understanding of the genetic composition of these important breeds in Brazil can help to reconstruct their history and open up perspectives for their future management and improvement of bovine production in the Brazilian tropical context.

Cattle were first introduced in Latin America by Spanish and Portuguese colonizers who brought taurine (*Bos primigenius taurus*) Iberian breeds in this part of the world [7]. Indicine breeds were imported from India during the 19th and 20th centuries, and it is estimated that a maximum of 7000 animals of indicine origin have been introduced in Brazil [8]. Thanks to their ability to adapt to the Brazilian tropical conditions, indicine cattle became popular and their population rapidly expanded up to the current numbers. This quick process was initiated by the use of locally available female cattle, such as Creoles that derive from Iberian cattle. Thereafter, repeated crosses with indicine males were used as a breeding strategy to recover pure indicine breeds [9]. The analysis of mitochondrial (mt) DNA haplotypes confirms this hypothesis. Brazilian indicine breeds possess the T1 and T3 taurine haplotypes that are very frequent in African and European taurine cattle, respectively [10], and present in Brazilian Creole and Iberian cattle breeds [11]. However, a Y-chromosome analysis of Brazilian cattle suggested an indicine paternal origin in the indicine breeds and indicine male introgression in the taurine creole populations [12]. Very few studies have analyzed the taurine introgression in Brazilian indicine cattle and most available reports are underpowered by the use of low numbers of microsatellite markers [13]. A recent study that was based on an unbiased panel of amplified fragment length polymorphism (AFLP) markers to investigate the genetic structure of several bovine populations at a global scale did not detect significant levels of taurine ancestry in three Brazilian breeds, including Nelore [14]. Several analyses using genome-wide single nucleotide polymorphisms (SNPs) have included Gir and Nelore individuals in their datasets [15-19], but in most cases with very few individuals (<20).

Here, we report a comprehensive analysis of the levels of genome-wide autosomal taurine admixture in Brazilian Nelore and Gir populations, through the use of dense SNPs and a comparison of the data with genotypes of other worldwide cattle breeds.

## Methods

Genotypes from the Illumina BovineHD BeadChip [20] (>777 K SNPs) were used. All samples were derived from previous studies, and with the exception of the Fleckvieh, Nelore and Gir breeds, individuals were chosen to represent the diversity within each breed. For the Fleckvieh, Nelore and Gir breeds, animals were selected to represent influential bulls that are widely used for artificial insemination in their respective breed. Nelore bulls representing two different types of breeding systems, pedigree and production, were included in equal proportions. Individuals (figures in brackets represent the number of individuals per breed) were sampled from five European taurine breeds: Holstein (67), Brown Swiss (73), Austrian Simmental Fleckvieh (96), Angus (37) and Hereford (27); one African taurine breed: N’Dama (48); and three indicine breeds: Brahman (35), Nelore (115) and Gir (100). The Nelore population included two groups of animals: 15 individuals that were considered as ancestral since they are first descendants of imported animals from India, and 100 individuals born after 2000 that represent the current population. All Gir individuals were also born after 2000 and represented the current population. Quality control of genotypes was performed within breed to exclude SNPs and individuals with more than 10% missing genotypes, and across all breeds to exclude monomorphic SNPs. After quality control, 697 115 autosomal SNPs and 28 (out of 343) mt SNPs were retained and used for analyses.

To obtain a general overview of the population structure, a multidimensional scaling analysis was performed by converting the genomic kinship coefficients from the identity-by-state (IBS) matrix generated with PLINK [21] to squared Euclidean distances between individuals via classical multidimensional scaling using the “cmdscale” function from R [22].The R script applied was cmdscale(as.dist(1-X), eig = TRUE), with X being the IBS full (upper, diagonal, lower triangle) matrix computed from PLINK. Genetic variability and differentiation of populations were also determined using Wright’s F-statistics, F_IS_ and F_ST_ [23,24].

Proportions of individual ancestry for K (number of assumed ancestral populations) ranging from 2 to 5 were evaluated using the unsupervised model-based approach implemented in ADMIXTURE v1.22 software [25]. The same analyses were run for a subset of the data after reducing the number of individuals per breed to a maximum of 20 randomly chosen animals, to evaluate the estimated ancestries using a balanced dataset for number of individuals per breed. We performed a small number of runs with different sets of random samples of 20 animals per group, with similar results. This is consistent with our experience from a different admixture study [26] in which large numbers of subsets of 10 animals per ancestral breed were used. The best ancestry estimates were obtained by using the cross-validation option implemented by ADMIXTURE. The mt SNPs were used to construct haplotypes [27] and the frequency for each identified haplotype was calculated per breed to evaluate the ability of these SNPs to separate individuals into indicine and taurine European or African clusters.

## Results and discussion

**Figure 1:**
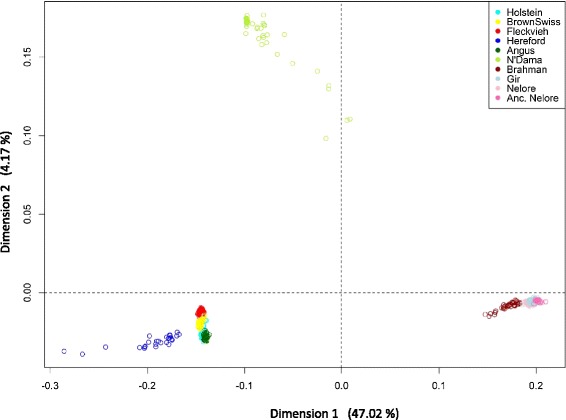
**Multidimensional scaling of all autosomal SNPs.** First (x-axis) and second (y-axis) dimensions with the variance explained shown in parenthesis under the corresponding axis separate taurine and indicine breeds and African and European taurine breeds.

F_ST_ values are in Table 1 and indicate very strong differentiation between indicine and taurine breeds. This is consistent with the results of multidimensional scaling (Figure 1). The first dimension explains almost half of the variance in the dataset and clearly separates indicine and taurine populations. The second dimension explains 4% of the variance and separates the African taurine N’Dama cattle from the European taurine populations. The Hereford breed shows a larger dispersion and is more distant from the other four European taurine breeds, which are tightly clustered, confirming previous results [16]. This separation of the Hereford breed probably reflects ascertainment bias of the SNPs since all SNPs of the Illumina BovineHD BeadChip were designed on the genome sequence that was derived from the sequence of a Hereford cow, thus increasing the observed diversity in this breed. A separate analysis excluding Hereford individuals (not shown) confirmed the separation between indicine, taurine and African taurine N’Dama breeds, with similar values (47, 81 and 4.48%, respectively). Dispersion in the N’Dama cluster towards the indicine gradient of positive PC1 coordinates reveals introgression of indicine genetic material in some individuals of this breed as reported by [17,18], and as shown by our results of ancestry estimation (see next paragraph). One hypothesis that may explain these levels of indicine ancestry is that the N’Dama cattle sampled here originate from different geographical locations in Nigeria, with the more pure African taurine populations possibly reflecting the result of selection against taurine/indicine crosses in the humid tsetse regions of West Africa as suggested by Freeman et al. [28]. The Nelore and Gir populations were clustered and the distance between these and the taurine breeds was greatest for the 15 ancestral Nelore individuals in the PC1 coordinate. The Brahman cluster was more dispersed and slightly closer to the taurine breeds, which agrees with the history of taurine introgression in this breed. The same patterns of separation between indicine and taurine cattle and between European and African taurine cattle explained by the first two components were reported in [16,18] in which the Illumina Bovine 50 K BeadChip and a larger number of breeds were used.

**Table 1:** **Indicators for population variability and differentiation (Wrights F**_**IS**_ **on diagonals and F**_**ST**_ **on off-diagonals)**

**Breeds**	**Holstein**	**Brown Swiss**	**Fleckvieh**	**Hereford**	**Angus**	**N‘Dama**	**Brahman**	**Gir**	**Nelore**	**Ancestral Nelore**
Holstein	−0.0058									
Brown Swiss	0.0906	−0.0258								
Fleckvieh	0.1368	0.0647	−0.0192							
Hereford	0.0683	0.1599	0.1368	0.0683						
Angus	0.0853	0.1037	0.0857	0.1609	0.0139					
N‘Dama	0.1631	0.1590	0.1419	0.2539	0.1885	0.0268				
Brahman	0.2707	0.2721	0.2014	0.3740	0.2863	0.2691	0.0137			
Gir	0.3105	0.3047	0.2933	0.4320	0.3366	0.2945	0.0477	−0.0097		
Nelore	0.2932	0.2916	0.2816	0.4062	0.3131	0.2845	0.0442	0.0475	−0.0063	
Ancestral Nelore	0.2948	0.2969	0.2864	0.3990	0.3097	0.2934	0.0488	0.0391	0.0023	−0.0101

**Figure 2:**
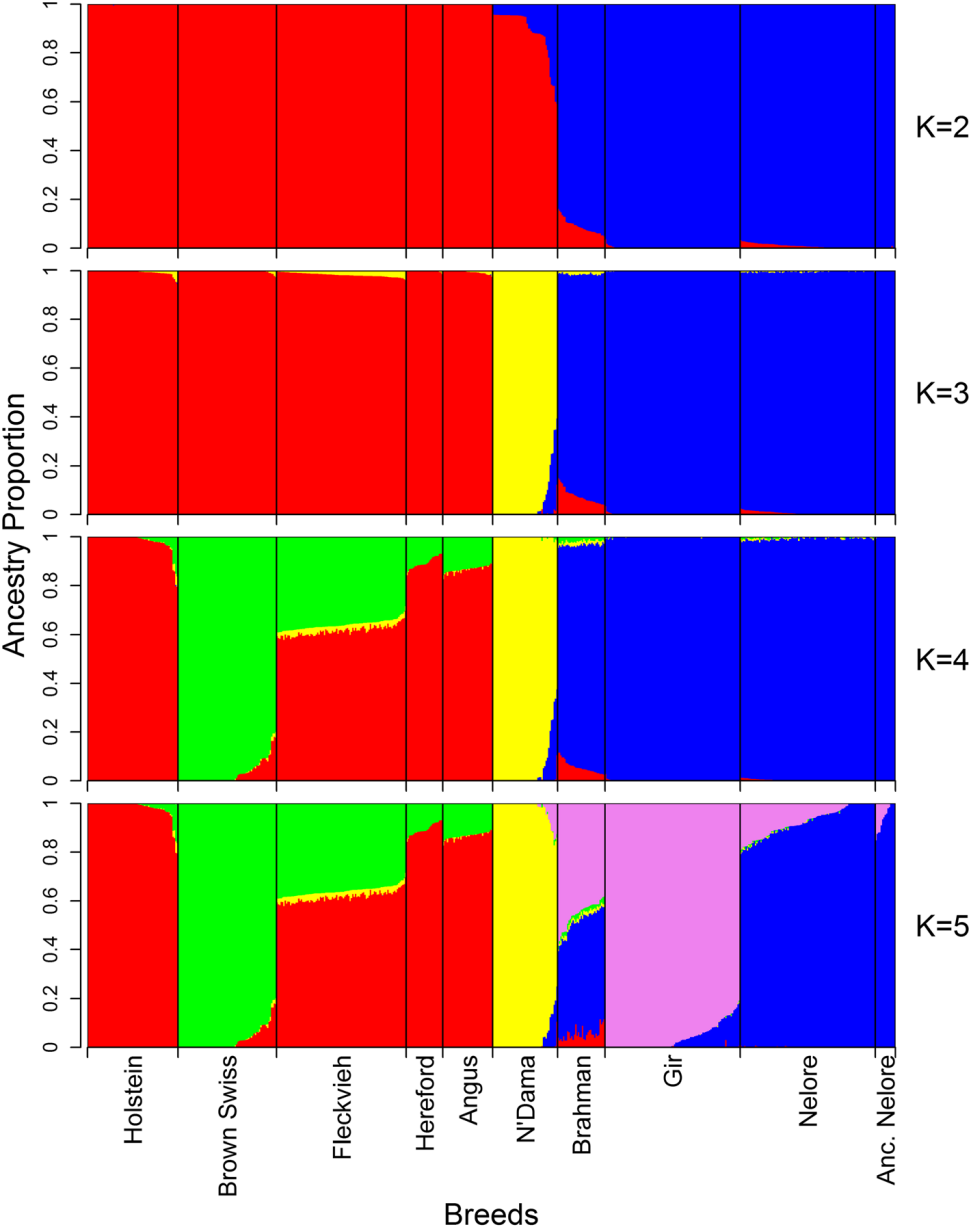
**Ancestry models with K ranging from 2 to 5 assumed ancestries.** Individual unsupervised model-based ancestry estimations for K ranging from 2 to 5 were assessed by ADMIXTURE. Individuals are represented by vertical bars, with breeds separated by black vertical lines and the proportion of each ancestry from 0 to 1 is shown on the y-axis, while breeds are indicated on the x-axis at the bottom of the K plots.

The results for the clustering of populations assuming two to five ancestries (K) are in Figure 2. Please note that the K range presented here was chosen arbitrarily for easier interpretation of ancestries. Please also note that the term ancestries used here represents statistical entities (clusters), not biologically separable units, thus the results need to be interpreted with caution. The first two estimated ancestries (K = 2) clearly separated taurine and indicine populations and showed that the Gir and Nelore breeds have an almost completely pure indicine autosomal ancestry with average levels of taurine introgression of 0.1% and 0.9%, respectively, while all ancestral Nelore individuals showed no signs of taurine ancestry. The Brahman sample exhibited a higher but still moderate taurine ancestry with an average level of taurine introgression of 8.9% across individuals, which is consistent with the known taurine introgression during the formation of this breed and with the results obtained by [15-18].

With K = 3, the African and European taurine populations were separated and levels of African taurine ancestry were low in some European populations, particularly in the Fleckvieh breed. A very low level of African taurine ancestry was estimated for the indicine populations, i.e. on average 1.5% in the Brahman and 0.4% in the Nelore populations. Indicine ancestry was observed in some of the African N’Dama individuals, but the number of these was smaller than with K = 2. Indeed with K = 3 a better fit of the model is probably obtained i.e. three ancestries are able to explain the larger divergence between African and European taurine ancestry as supported by the findings of Decker et al. [17].

With K = 4, Brown Swiss and Holstein cattle were clearly separated, with the other three European populations showing intermediate levels of both ancestries. With the addition of one more assumed ancestry (K = 5), the Gir and Nelore breeds were separated into two clusters of indicine ancestry and the Brahman breed had intermediate levels of taurine and indicine ancestries. The separation of the Nelore and Gir breeds into different ancestries is consistent with the fact that they originated from separate Indian populations; the first population derived from gray breeds of Northern and central India, and the second one from the red and white-speckled cattle from the West coast of India, south of the Kathiawar peninsula [29]. The results obtained for the Brahman breed reflect the historical records on the formation of this breed, which indicate that a synthetic indicine population was created in the United States during the early 1900’s by breeding indicine animals of the Nelore, Gir and Guzerat breeds imported mainly from Brazil to intensively upgrade the available taurine cattle from a base population [29,30]. Levels of taurine introgression were higher in the Brahman breed than in the Nelore and Gir breeds, which may indicate the preservation of taurine specific haplotypes through stronger selection for specific productive characteristics as suggested by Bolormaa et al. [31]. Previous analyses based on mt DNA sequence data also confirmed a maternally-derived taurine influence in Brahman cattle since both European and African characteristic mt DNA haplogroups were found in animals from this breed [12].

When the same analyses were repeated by restricting the number of individuals per breed to a maximum of 20 randomly chosen animals [see Additional file 1], the results differed most with K = 3. European taurine breeds displayed higher levels of African taurine ancestry than those in the full dataset, with the highest level (18%) observed for individuals of the Fleckvieh breed. In addition, the analysis with K = 4 separated the Hereford from the other European taurine breeds, which is more consistent with the results obtained from multidimensional scaling. The lowest cross-validation error for different numbers of ancestries was obtained with 10 assumed ancestries (results not shown) for which each breed was assigned a main ancestry: the N’Dama breed was separated in two different ancestries based on the observed indicine introgression at K values lower than 10, and all Nelore individuals including the ancestral individuals were assigned to a single cluster.

Analysis of mt SNPs led to the reconstruction of 27 haplotypes and their frequencies are summarized in Table 2. Our results indicate that the mt SNPs included in the Illumina BovineHD BeadChip could neither separate the analyzed populations assayed nor attribute haplotypes to the known mt haplogroups. The strongest evidence came from one individual of pure indicine origin (ancestral Nelore individual) that was assigned the most frequent haplotype across all taurine breeds (Haplotype 1), while one Holstein and one Hereford individual were each assigned the haplotype that had the highest frequency in the ancestral Nelore individual (Haplotype 2). Mitochondrial DNA analyses for the characterization of bovine haplogroups have widely used a major hypervariable region in the mt D-loop, located in the bovine mt genome between 16 023 and 16 262 bp [32]. Although eight SNPs were located within this region, seven were monomorphic, and consequently non-informative for the individuals studied. A haplotype separation approach was also undertaken using the nine Y-chromosome SNPs that remained after quality control, but it did not reveal any separation between indicine and taurine haplotypes (results not shown).

**Table 2:** **Frequency of estimated mitochondrial haplotypes for each breed**

**Breed**	**Haplotype**	**1**	**2**	**3**	**4**	**Others**
Holstein		0.91	0.02	-	-	0.07
Brown Swiss		0.77	-	0.12	-	0.11
Fleckvieh		0.82	-	-	0.01	0.17
Hereford		0.93	0.04	-	-	0.03
Angus		0.86	-	-	0.03	0.11
N’Dama		0.90	-	0.06	-	0.04
Brahman		0.54	0.17	-	0.14	0.15
Gir		0.84	0.15	-	-	0.01
Nelore		0.96	0.03	-	-	0.01
Ancestral Nelore		0.08	0.85	-	-	0.07

Rounded estimated frequencies for haplotypes with frequencies higher than 0.05 and sum of all other observed haplotypes in each breed.

The main objective of this study was to assess the level of admixture in current Nelore and Gir Brazilian populations. Taurine genome admixture events during the initial expansion of these two breeds are reported in historical records and supported by published results on mt DNA. High-resolution genome-wide analyses indicate that the individuals in the current populations of both breeds possess levels of autosomal taurine ancestry lower than 1%, which is consistent with a process of several decades of continuous purifying selection through the use of indicine imported males as suggested by [9]. Assuming a strict upgrading system from a pure Creole population, seven generations would be required to achieve the observed levels of taurine introgression, which considering a generation interval of 7 to 8 years [33] would correspond to between 50 and 65 years. This is reasonable scenario for both Brazilian breeds, for which official pedigree recording was established in 1936 [3].

The taurine ancestry observed in both Brazilian breeds derives from individuals that came from both Europe and Africa. This confirms that the Brazilian Creole breeds are likely the source of the introgression. In fact, the South American Creole populations have been reported to have a moderate level of African taurine ancestry [17] and to be descendants of Spanish and Portuguese cattle that carry mt haplotypes that are frequently found in African taurine populations [10,12]. It is worth noting that the sample of Nelore individuals analyzed here included in equal proportions individuals that are registered as being of pure indicine origin (“PO” in the national registry) and individuals not considered pure by registry, but no difference in the levels of admixture were observed among these two groups of Nelore individuals.

## Conclusions

Using a high-resolution genome-wide DNA analysis, we identified very low levels of taurine introgression in Brazilian Nelore and Gir cattle populations, which contradicts the previous observations in [9,13] but supports those in [19]. Our findings indicate that the current Brazilian Nelore and Gir populations are of almost pure indicine ancestry regarding their autosomal genome. The Brahman population used in this analysis showed average levels of taurine ancestry of 9%, which is consistent with the fact that taurine animals were used to develop this breed in the USA. This result also suggests that, in this breed, there has been a stronger selection for production characteristics that derive from the influence of taurine haplotypes. The Nelore and Gir individuals that were genotyped in this study are all bulls used for artificial insemination and reflect the top of the breeding pyramid in these two breeds. Random sampling of animals from production herds across the country would provide a more complete picture and would be useful to evaluate admixture levels in commercial populations. Finally, the mt SNPs available in the Illumina BovineHD BeadChip could not differentiate between the major known mt haplogroups and could not identify subspecies or subpopulation specific haplotypes among the breeds analyzed.

## Additional file

Additional file 1:
**Ancestry models with K = 2 to 5 assumed ancestries for the dataset reduced to a maximum of 20 individuals per breed.** Individual unsupervised model-based ancestry estimations for K ranging from 2 to 5 assessed by ADMIXTURE using a reduced dataset with a maximum of 20 individuals per breed chosen at random from the complete dataset. Individuals are represented by vertical bars, with breeds separated by black vertical lines, and the proportion of each ancestry from 0 to 1 is shown on the y-axis, while breeds are indicated on the x-axis at the bottom of the K plots.
