# Supplementary figures and images for: Low levels of taurine introgression in the current Brazilian Nelore and Gir indicine cattle populations
Source: Genet Sel Evol. 2015 Apr 17;47(1):31. doi: 10.1186/s12711-015-0109-5 (PMC4404172; doi:10.1186/s12711-015-0109-5)

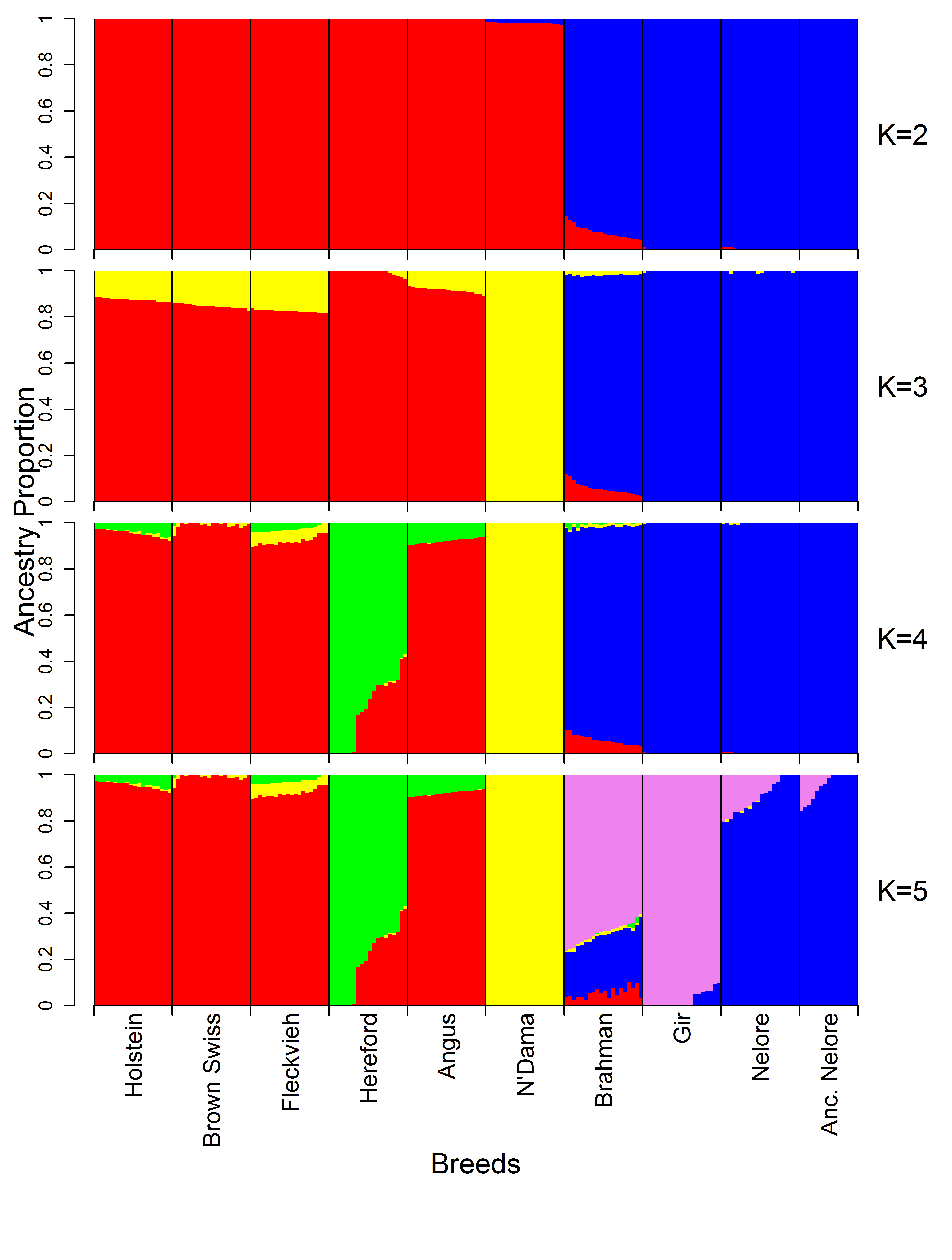

Supplement: Additional file 1: — Ancestry models with K = 2 to 5 assumed ancestries for the dataset reduced to a maximum of 20 individuals per breed. Individual unsupervised model-based ancestry estimations for K ranging from 2 to 5 assessed by ADMIXTURE using a reduced dataset with a maximum of 20 individuals per breed chosen at random from the complete dataset. Individuals are represented by vertical bars, with breeds separated by black vertical lines, and the proportion of each ancestry from 0 to 1 is shown on the y-axis, while breeds are indicated on the x-axis at the bottom of the K plots. [file 12711_2015_109_MOESM1_ESM.png]
